# Low Match Between the Emerging ‘Mayotte-like’ Strain and the 919-Strain Bovine Ephemeral Fever Vaccine

**DOI:** 10.3390/vaccines14090830

**Published:** 2026-09-21

**Authors:** Natalia Golender, Dan Gleser, Gabriel Kenigswald, Shani Scheinin, Eyal Klement

**Affiliations:** 1 Koret School of Veterinary Medicine, The Robert H. Smith Faculty of Agriculture, Food & Environment, The Hebrew University of Jerusalem, P.O. Box 12, Rehovot 761001, Israel; dan.gleser@mail.huji.ac.il; 2 Department of Virology, Kimron Veterinary Institute, P.O. Box 12, Bet Dagan 5025001, Israel; 3 Hachaklait Veterinary Services, P.O. Box 3039, Caesarea 3088900, Israel; kenigswald@hak.org.il (G.K.); scheinin@hak.org.il (S.S.)

**Keywords:** vaccine effectiveness, neutralizing antibodies, viral diseases of cattle, bovine ephemeral fever, arbovirus, cross reactivity, *Rhabdoviridae*, serologic tests, neutralization tests

## Abstract

**Background/Objectives**: Bovine ephemeral fever virus (BEFV) causes an economically important arthropod-borne cattle disease. In 2023, a lineage I “Mayotte-like” (ML) strain emerged in Israel during an outbreak in vaccinated herds, raising concerns about its antigenic match with the live-attenuated vaccine based on Australian strain 919. We compared G-protein neutralizing sites and cross-neutralization among the vaccine strain, Israeli lineage IIIa strains from 2000 and 2021, and ML. **Methods**: G1–G3 neutralizing-site sequences were compared. Serum-neutralization assays tested all four viruses using sera from 87 cattle across five vaccination/exposure groups. Log_2_-transformed titers were compared within animals. **Results**: ML differed from the vaccine strain at eight amino acid positions within G1–G3, whereas the lineage IIIa strains differed from the vaccine strain at three or four positions. In vaccinated, unexposed calves, neutralizing titers against the vaccine and 2021 strains were 4.0- and 2.7-fold higher, respectively, than against ML (both *p* < 0.01). In unvaccinated 2021-exposed cattle, titers against the 2021 strain were 6.5-fold higher than against ML (*p* < 0.01). In unvaccinated 2023-exposed cattle, titers against ML were 4.76-fold higher than against the vaccine strain (*p* < 0.01). Between-strain differences were smaller in vaccinated 2023-exposed cattle. **Conclusions:** Sequence divergence and cross-neutralization indicate a markedly reduced antigenic match between the Australian 919 vaccine strain and the ML strain. Given the continued circulation and emergence of antigenically divergent BEFV strains and their potential for geographic spread, challenge and field-effectiveness studies are urgently needed to determine the clinical protection afforded by the current vaccine. Lineage-matched or multivalent vaccines should therefore be prioritized to broaden protection against circulating and newly emerging strains.

## 1. Introduction

Bovine ephemeral fever (BEF) is a viral disease of animals from the family *Bovidae*, mostly cattle and buffalo. The morbidity rate in susceptible cattle populations is usually high (up to 80–100%), while the mortality in most cases rarely exceeds 1% [1]. The disease is characterized by rapid onset and rapid recovery. The duration of clinical signs is usually short, lasting only 1–3 days, characterized by biphasic acute hyperthermia, lameness, and ocular and nasal discharge before recovery [2]. In more seriously affected cattle, following the acute phase of infection, symptoms such as recumbency, synovitis, lethargy, muscle stiffness, lameness, reluctance to move, inappetence, anorexia, mortality, and, in rare cases, neural signs, such as prolonged paralysis and ataxia, may be observed. 

BEF is transmitted by blood-sucking insects. The disease predominantly occurs during the wet season in tropical regions and during summer and early autumn in subtropical or temperate areas, coinciding with conditions that promote the multiplication of biting insects [3]. In endemic areas, the disease has been recorded every 2–4 years [3,4]. BEF has been reported in Africa, Asia, and Australia, as well as in the Far and Middle East [2,3,4,5,6,7,8,9]. Recently, non-recognized strains of BEFV were isolated in Mayotte, China, India, and Israel [4,7,8,9].

During the last two decades, an increasing frequency and severity of the disease, with alarmingly high case-fatality rates, sometimes exceeding 20%, have been reported in China and Turkey [10,11,12]. The major economic impact caused by BEF in dairy cattle arises from reduced milk production, mortality, and culling of severely affected animals in the dairy industry. In the feedlot cattle industry, economic losses are associated with reduced production, temporary infertility in males, and the temporary disablement of draught animals [1]. 

BEFV is a member of the genus *Ephemerovirus* in the family *Rhabdoviridae*, which possesses a single-stranded, negative-sense RNA genome coding for five structural proteins (N, P, M, G, and L), one nonstructural protein (GNS), and multiple smaller accessory proteins. The primary immune response that neutralizes BEFV targets specific antigenic determinants located in three major distinct antigenic sites (G1–G3) on the envelope G protein [2,13]. Regarding the phylogenetic relationship, a recent study by Golender et al. [4] reported that BEFV constitutes a single serotype, which can be subdivided into four lineages. 

Vaccination is the predominant strategy for preventing the disease [14]. Various platforms have been employed to produce BEFV vaccines, with inactivated and live-attenuated vaccines being the most common. A widely used, long-standing commercial live-attenuated vaccine, based on the 919 Australian isolate, was evaluated a few decades ago in a field trial. The study demonstrated a high vaccine effectiveness of 90% after two doses, lasting for at least 12 months [15]. This vaccine was recently evaluated under its current commercial trade name, Ultravac^®^, produced by Zoetis^TM^. In this recent study, the vaccine effectiveness (VE) was estimated as 60% for two doses when administered shortly before the emergence of the BEF outbreak [16]. The intermediate-term dose-effectiveness of the vaccine was further evaluated in a retrospective cohort study. The study demonstrated a VE of 82% for four doses, 66% for three doses, and 39% for two doses. Based on these findings, it was recommended to vaccinate calves for the first time at four to six months of age, with two doses administered one month apart, followed by a third dose and optionally a fourth dose—six to twelve months later, ideally timed before the high-risk season [17].

From 2000 to 2021, all strains identified in Israel belonged to lineage IIIa. However, in 2023, novel BEFV strains from lineage I (Mayotte-like strains) emerged in Israel for the first time [4]. The outbreak spread widely across dairy farms, with clinical signs consistent with BEF, including fever, reduced milk production, lameness, and recumbency. Notably, this outbreak affected several farms with vaccinated cattle, raising concerns about the ongoing effectiveness of the widely used Ultravac^®^ vaccine against this antigenically distinct strain. Moreover, the ‘Mayotte-like’ strains exhibit antigenic differences from previously circulating local strains in Israel, reinforcing concerns regarding the effectiveness of existing vaccines, such as the Ultravac^®^ vaccine, against this emerging variant. To our knowledge, there is very limited data on antigenic matching between BEFV strains from different lineages, and no data yet published on the antigenic matching of the ‘Mayotte-like’ strain with other circulating and vaccine strains. Notably, Kato et al. demonstrated that antigenic differences between lineages can result in serum-neutralization titers lower by 4–16-fold compared to homologous strains [18]. 

We hypothesized that the 2023 Mayotte-like lineage I strain is antigenically less well matched to the Australian 919 vaccine strain than the previously circulating Israeli lineage IIIa strains, resulting in reduced cross-neutralization by vaccine-elicited and lineage IIIa exposure-elicited sera. To test this hypothesis, we compared amino acid variation within the G-protein neutralizing sites and reciprocal serum-neutralization responses among two Israeli lineage IIIa strains, the 2023 Mayotte-like lineage I strain, and the Australian 919 vaccine strain. Our findings provide insights into the vaccine’s potential to confer protection against emerging strains and may guide future vaccine development strategies.

## 2. Materials and Methods

### 2.1. Vaccination

In both the field trial and for routine vaccination, a commercially available live-attenuated vaccine was used (Ultravac® BEF Vaccine (Zoetis Australia Pty Ltd., Rhodes, NSW, Australia)), based on the Australian 919 strain (also known as strain BB7721, accession number U04166), which belongs to lineage IV. All vaccinated animals received at least two doses administered one month apart.

### 2.2. Study Design

Five different groups of positive serum samples were selected: **‘vac-21’**: non-infected calves that were vaccinated during a field trial, which took place in 2021; **‘exp-21’**: non-vaccinated calves, which exhibited BEF typical clinical signs during a BEFV outbreak in 2021 (lineage IIIa); **‘vac&exp-21’**: vaccinated calves and cows, which exhibited BEF typical clinical signs during a BEFV outbreak in 2021 (lineage IIIa); **‘exp-23**‘: non-vaccinated calves and cows, which exhibited BEF typical clinical signs during the BEFV outbreak in 2023 caused by the Mayotte-like BEFV strain (lineage I); **‘vac&exp-23’**: vaccinated calves and cows, which exhibited BEF typical clinical signs during the BEFV outbreak in 2023. Since most of the animals in the latter two groups are cows, they may have been exposed to BEFV in 2021 as well. Information on the animals’ groups is presented in Table 1.

### 2.3. Sequencing and Sequence Analyses of BEFV Strains Used in the Study

The ISR-1512/23 BEFV strain was partially sequenced by the Sanger method with overlapping genome fragments (primer sequences are presented in Table S1). The cDNA fragments were purified with the MEGAquick-spin Total Fragment DNA Purification Kit (iNtRON Biotechnology, Seongnam-si, Gyeonggi-do, Republic of Korea) and subsequently sequenced at Hylabs, Rehovot, Israel. Sequencing was performed in both directions using standard Sanger methods on an ABI 3730xl DNA Analyzer (Thermo Fisher Scientific, Waltham, MA, USA). Whole-genome coding sequences of the BEFV vaccine strain 919 and the Israeli Yakum-2000 strain were included in the analysis (GenBank accession numbers PQ619394 and PQ619395, respectively). The G1–G3 neutralizing sites were defined according to the epitope mapping of Kongsuwan et al. [19]. Assembly of nucleotide (nt) sequences of the viruses was performed using Geneious version 9.0.5 (Biomatters, Auckland, New Zealand), while alignment was done with BioEdit programs (https://bioedit.software.informer.com/7.2/, accessed on 19 September 2026). All assembled sequences were submitted to NCBI GenBank.

### 2.4. Phylogenetic and Pairwise Sequence Analyses

Evolutionary analyses were conducted in MEGA X [20]. Pairwise analysis of nt sequences was done with the BioEdit program, while for pairwise analysis of amino acid (aa) sequences, MEGA X (Version 10) was used.

### 2.5. BEFV Propagation and Titration

Four different BEFV strains were propagated and titrated in Vero cells (origin: green monkey kidney cells): The Australian vaccine Ultravac^®^ (**strain 919**, also known as BB7721 strain), **Yaqum-2000**, Mayotte-like isolate **ISR-1512/23** (acc. no. PQ409321), and **ISR-3180/1/21** (acc. no. OQ171228). The 50% tissue culture infectious dose (TCID_50_) was calculated according to the Reed–Muench method [21]. 

Viruses were propagated in Vero cells at 37 °C in a humidified atmosphere with 5% carbon dioxide. The Vero cells were cultured in Dulbecco’s Modified Eagle Medium (DMEM), supplemented with 1% streptomycin–penicillin–amphotericin ready-to-use solution (Pen-Strep-Ampho. B, Biological Industries, Beit Haemek, Israel). The medium was further supplemented with either 10% fetal calf serum (FCS) (growth medium) or 2% FCS (maintenance medium) (Sigma-Aldrich, St. Louis, MO, USA).

### 2.6. Serum-Neutralization Test (SN)

Serum samples were serially diluted two-fold in maintenance medium using double-line 96-well cell culture U-bottom microplates, starting with an initial dilution of 1:4. Sera dilutions were mixed with equal volumes of 100 TCID_50_/0.05 mL of virus and incubated at 37 °C for 1 h. Following incubation, the serum/virus mixtures were transferred to 96-well cell culture flat-bottom microplates containing a 24 h monolayer of Vero cells. The plates were incubated for 30 min, after which 0.1 mL of maintenance medium was added to each well. The results were read after 5 days of incubation under standard cell culture propagation conditions.

The geometric mean titer (GMT) for each cross-group serum-neutralization test (SN) with different combinations of BEFV strains was calculated as the mean of the log2-transformed titer ratios. Serum dilutions were extended up to 1:4096, corresponding to a maximum GMT of 12. The TCID50 was calculated for each BEFV strain. The TCID_50_ values were as follows: 10^7.47^ for the Australian strain BB7721 (acc. no. PQ619394), 10^7.21^ for the Yaqum-2000 strain (acc. no. PQ619395), 10^7.3^ for ISR-1512/23 (acc. no. PQ409321), and 10^5.3^ for ISR-3180/1/21 (acc. no. OQ171228).

### 2.7. Statistical Analyses and Data for Visualization 

For each serum sample, pairwise differences in log_2_-transformed serum-neutralization titers were calculated for all six comparisons among the four BEFV strains. Within each exposure group, the distribution of the paired differences was assessed for normality using the Shapiro–Wilk test. Comparisons for which there was no evidence of departure from normality (*p* ≥ 0.05) were evaluated using a two-sided paired-samples *t*-test, whereas comparisons with non-normally distributed paired differences (*p* < 0.05) were evaluated using the two-sided Wilcoxon signed-rank test. Mean paired differences, standard errors (SEs), and 95% confidence intervals were calculated for descriptive presentation. Statistical analyses were performed using Python 3.12.14 with pandas 2.2.3, NumPy 2.3.5, and SciPy 1.17.0; figures were generated using Matplotlib 3.10.8. Horizontal jitter was applied to individual data points for visualization only and did not affect the statistical calculations.

## 3. Results

### 3.1. Comparison of Amino Acid Composition of G-Protein Antigenic Sites

**Figure 1:**
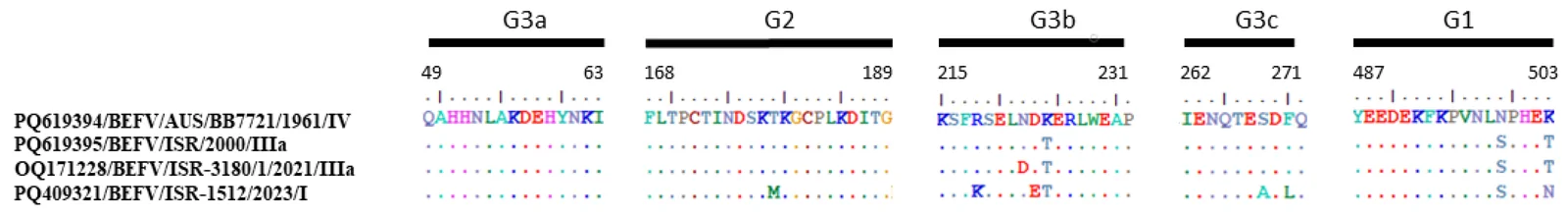
Alignment of the amino acid (aa) sequences corresponding to the G1, G2, and G3 neutralizing sites of the BEFV G protein of the Israeli and vaccine BEFV strains used in the serum-neutralization tests. Substitution residues that differ from the top sequence are highlighted. The analyzed BEFV strains are identified by their accession number/country/strain/year of isolation/lineage.

The alignment of G1–G3 neutralizing sites for all four BEFV strains used in the SN tests is presented in Figure 1. The Ultravac^®^ vaccine seed strain (Australian BEFV strain 919, lineage IV) differed at these sites by three and four amino acids from the Israeli Yakum-2000 and ISR-3180/1/21 strains (both lineage IIIa), respectively, and by eight amino acids from the Mayotte-like ISR-1512/23 strain (lineage I). Thus, the Mayotte-like strain exhibited the greatest sequence divergence from the vaccine strain within the known neutralizing sites.

### 3.2. Phylogenetic and Pairwise Sequence Comparisons

The information about nt and aa sequence identity presented in Table 2 indicates that ISR-1512/23 (‘Mayotte-like’) is more divergent from the vaccine strain than are the Israeli strains isolated in 2000 and 2021. Consistently, the two local Israeli strains are more similar to the vaccine strain than to ISR-1512/23 (‘Mayotte-like’).

**Table 2:** Pairwise nucleotide and amino acid sequence identities (%) among the BEFV strains used in serum-neutralization tests (AUS, Australian vaccine strain 919; ISR_2000, Israeli 2000 strain (Yakum-2000); ISR_2021, Israeli 2021 strain; ISR_2023, Israeli 2023 strain, ‘Mayotte-like’).

Strain	AUS	ISR_2000	ISR_2021
**AUS**	-		
**ISR_2000**	90.60/94.56	-	
**ISR_2021**	90.07/94.20	98.34/98.63	-
**ISR_2023**	86.42/92.75	87.44/93.84	87.27/93.48

**Figure 2:**
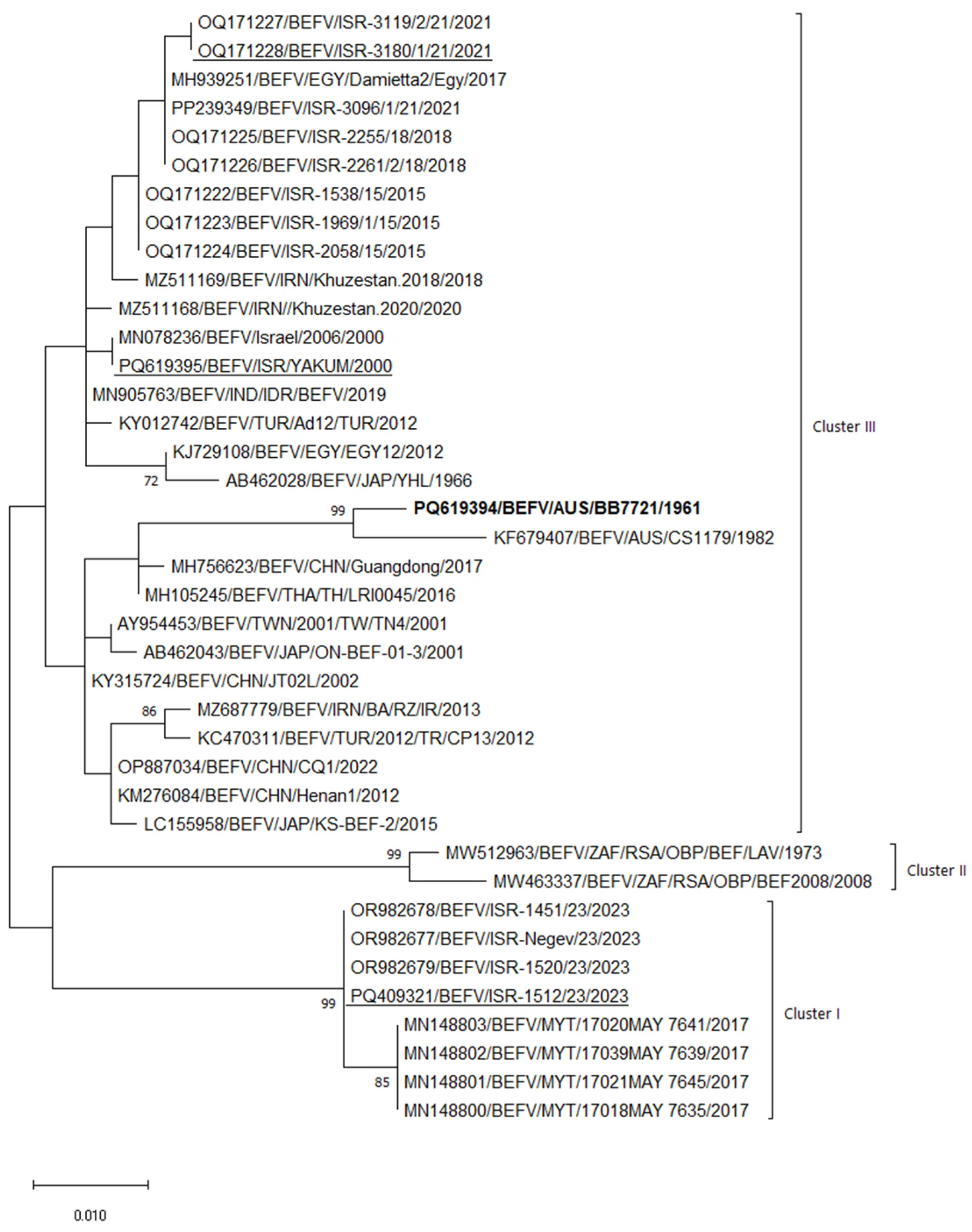
Phylogenetic tree of Israeli and global bovine ephemeral fever viruses (BEFV) analyzed by amino acid sequences of the glycoprotein. Israeli BEFV strains used for serum-neutralization tests are underlined, while the vaccine strain is shown in bold. The phylogeny was inferred using the Maximum Likelihood method and the JTT matrix-based model method. The percentage of replicate trees in which the associated taxa clustered together in the bootstrap test (1000 replicates) is shown next to the branches. Viruses were identified by accession number/virus species/location/isolate/year. The tree is drawn to scale, with branch lengths measured in the number of substitutions per site.

Phylogenetic analysis of BEFV strains based on aa sequences of the G protein revealed differentiation into three main clusters (Figure 2). The 1st cluster consists of BEFV strains belonging to the Mayotte and Israeli ‘Mayotte-like’ strains. The 2nd cluster includes BEFV strains identified in South Africa, while the 3rd cluster contains two subclusters, which comprise Asian and Australian BEFV strains. However, according to the phylogenetic tree, Australian strains share a common ancestor with BEFV strains from the Far East. The phylogenetic tree highlights the significant divergence of Asian and Australian strains.

### 3.3. Cross-SN of Different Groups of Cattle Sera

**Figure 3:**
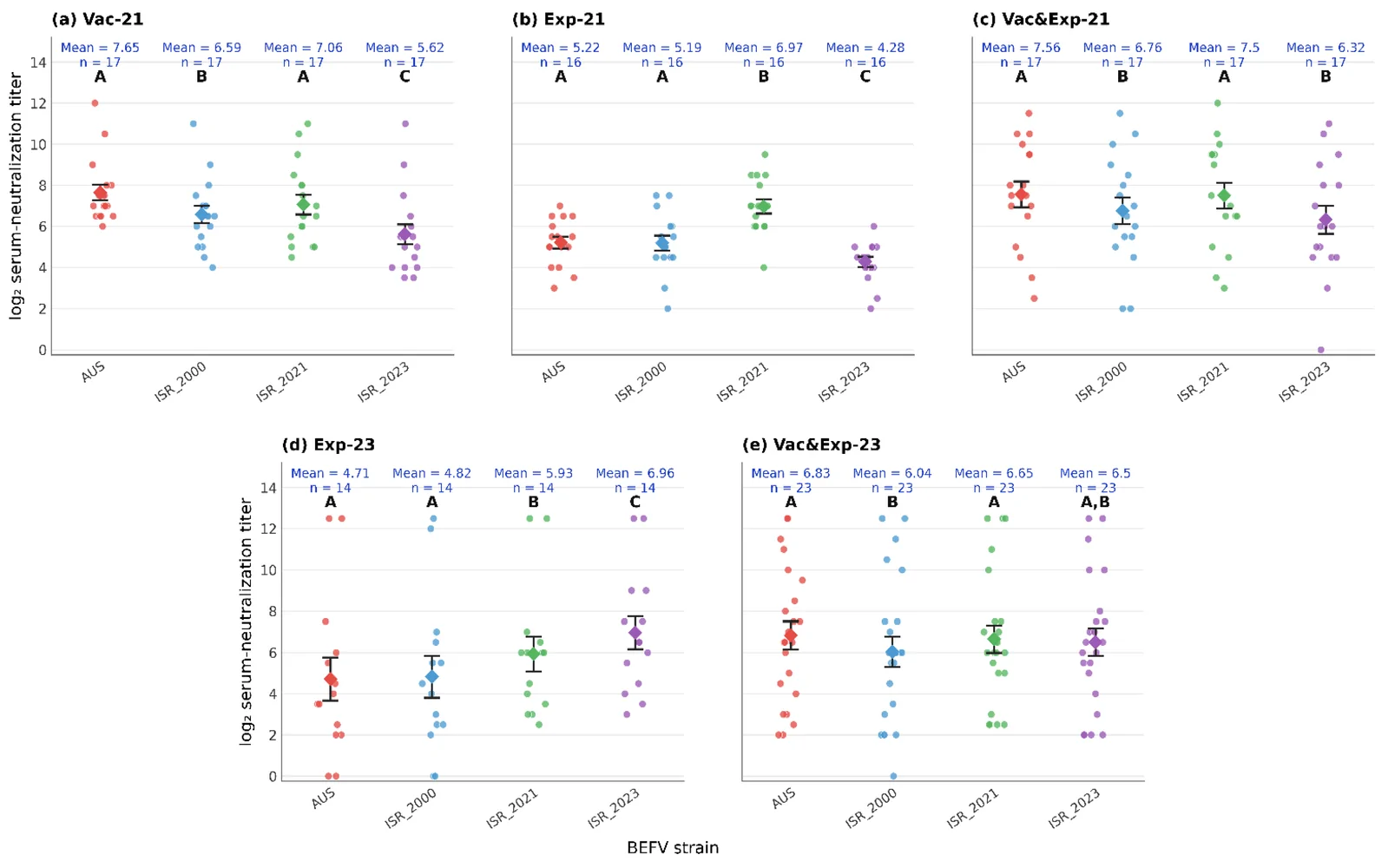
Serum-neutralization titers against four bovine ephemeral fever virus strains in five serum groups. Panels show sera from: (**a**) vaccinated animals without recorded natural exposure sampled in 2021 (Vac-21); (**b**) unvaccinated animals clinically affected during the 2021 outbreak (Exp-21); (**c**) vaccinated animals clinically affected during the 2021 outbreak (Vac&Exp-21); (**d**) unvaccinated animals clinically affected during the 2023 outbreak (Exp-23); and (**e**) vaccinated animals clinically affected during the 2023 outbreak (Vac&Exp-23). Colored circles represent individual log_2_-transformed serum-neutralization titers. Diamonds and error bars represent the arithmetic mean and standard error (SE), respectively. The mean and number of tested sera (n) are displayed above each strain. Capital letters represent the results of within-group paired-samples comparisons: strains sharing at least one letter were not significantly different, whereas strains with no letter in common differed significantly (*p* < 0.05). AUS, Australian 919 vaccine strain; ISR_2000, Israeli Yakum-2000 strain; ISR_2021, Israeli ISR-3180/1/21 strain; ISR_2023, Israeli Mayotte-like ISR-1512/23 strain; BEFV, bovine ephemeral fever virus.

Geometric mean titer (GMT) (i.e., log2-transformed titers) was calculated for each of the five exposure groups against each of the four tested viruses (Figure 3a–e). The statistical differences between the neutralizing titers are depicted in Table 3 and Figure S1a–e. Among the sera of vaccinated animals (Vac-21), the highest GMT (7.65) was observed against the homologous Australian 919 vaccine strain and the lowest GMT (5.62) was observed against the 2023 emerging strain (Figure 3a), with a mean paired difference of 2.03 log2 units (*p*-value < 0.01) (Table 3, Figure S1a). This indicates that the 2023 emerging strain isolate was neutralized four-fold less efficiently than the vaccine strain by antibodies elicited through vaccination. The GMT against the 2021 circulating strain isolate was 7.06, representing a mean paired difference of 1.44 log2 units, corresponding to a 2.7-fold difference when compared with the emerging 2023 strain GMT (GMT- 5.62, *p* < 0.01; Table 3, Figure S1a). These findings demonstrate that the vaccine strain was substantially better matched with the Middle Eastern BEFV strain circulating in 2021 than with the ‘Mayotte-like’ strain circulating in 2023. In sera collected from unvaccinated calves exposed to BEFV during the 2021 outbreak (Exp-21), the GMT against the homologous 2021 circulating strain, isolated during the outbreak, was 6.97. This titer was significantly higher than the GMT against the emerging 2023 strain (4.28), with a mean paired difference of 2.69 log2 units, corresponding to a 6.5-fold difference (*p*-value < 0.01) (Table 3; Figure 3b and Figure S1b). These findings provide evidence of substantial antigenic divergence between the 2021 circulating strain and the 2023 ‘Mayotte-like’ emerging strain. This supports the broader evident distinction between Middle Eastern strains circulating prior to and after the ‘Mayotte-like’ strain emergence in 2023.

Among sera collected from vaccinated animals exposed to BEFV during the 2021 outbreak (Vac&Exp-21), the highest GMT (7.56) was observed against the vaccine strain and thereafter the 2021 strain with an approximately similar GMT. In contrast, the lowest GMT (6.32) was recorded against the 2023 emerging strain, yielding a mean paired difference of 1.24 and 1.18 log2 units, respectively (*p* < 0.01; Table 3; Figure 3c and Figure S1c). 

The group of unvaccinated cows exposed to BEFV in 2023 (Exp-23) demonstrated the highest GMT of 6.96 to the homologous 2023 emerging strain, with a mean paired difference of 2.25 (*p*-value < 0.01) compared with the vaccine strain GMT of 4.71, corresponding to a 4.76-fold difference (Table 3, Figure 3d and Figure S1d). The relatively high GMT against the 2021 circulating strain (5.93) may reflect prior exposure of some animals to the BEFV strain circulating in 2021, as this group comprised mature cows that were old enough to have been exposed during that outbreak.

The differences in GMTs among the tested strains were smallest in cows that had been both vaccinated and exposed to BEFV during the 2023 outbreak (Vac&Exp-23; Figure 3e and Figure S1e). This finding may reflect a broader neutralizing-antibody repertoire generated by vaccination and potential natural exposure to both the 2021 and 2023 BEFV strains.

## 4. Discussion

The 2023 epidemic in vaccinated dairy herds in Israel, caused by a lineage I ‘Mayotte-like’ BEFV strain, raised concern regarding the effectiveness of the Ultravac^®^ BEF, 919 strain-based vaccine for protection against it. To investigate this, in the current study, we determined the aa differences in neutralizing antigenic sites and antibody cross-neutralization (and hence cross-protection) among this emerging strain, previously circulating Middle Eastern lineage IIIa strains, and the Australian 919 vaccine strain.

We show that the lineage I strain exhibits substantial antigenic differences from the lineage IIIa strains that previously circulated in the region. Notably, sequence comparison identified eight aa substitutions within the neutralizing antigenic sites of the G protein between the Australian 919 vaccine strain and the ‘Mayotte-like’ strain, whereas the vaccine strain differs from previous Middle Eastern lineage IIIa viruses by only three to four aa. This greater divergence could contribute to reduced vaccine-induced protection against the BEFV strain that emerged in 2023. The serological findings further support this concern. Neutralizing titers in vaccinated calves were 4.0- and 2.7-fold higher against the vaccine strain and the 2021 Middle Eastern strain, respectively, than against the ‘Mayotte-like’ strain. Moreover, sera from unvaccinated cows naturally exposed during the 2023 outbreak yielded a GMT against the homologous ‘Mayotte-like’ strain that was 4.76-fold higher than that against the vaccine strain. Together, these reciprocal cross-neutralization patterns provide evidence of substantial antigenic mismatch between the Australian 919 vaccine strain and the emerging ‘Mayotte-like’ strain. 

Historical experimental studies demonstrated antigenic differences among BEFV strains through cross-neutralization in cattle [22]. Previously, cattle in Israel were vaccinated with an inactivated vaccine based on the Yakum-2000 strain. During the 2008 outbreak, this vaccine demonstrated limited effectiveness of approximately 50%, observed only after three doses [23]. The Israeli vaccine was subsequently replaced in Israel by the commercial live-attenuated vaccine based on the Australian 919 strain, currently marketed by Zoetis as Ultravac^®^ BEF. According to research conducted four decades ago, administration of two doses of this vaccine in Australia demonstrated 90% vaccine effectiveness in preventing clinical disease for at least 12 months [24]. Recent evidence indicates that the effectiveness of Ultravac^®^ against the Middle Eastern lineage IIIa strains is substantially lower [16,17]. Although our in vitro findings do not directly measure clinical protection, this mismatch raises concern that the 919 strain-based vaccine may be even less effective in preventing clinical disease caused by the emerging strain.

Vaccine-strain mismatch is well documented among RNA viruses affecting animals and humans, including influenza, Newcastle disease, and foot-and-mouth disease viruses [25,26,27]. Similar vaccine–virus mismatch has been documented among arboviruses, including diminished neutralization of re-emerging genotype V Japanese encephalitis virus by genotype III-based vaccines, reduced dengue vaccine efficacy against genetically mismatched circulating viruses, and incomplete or unpredictable heterologous protection between bluetongue virus serotypes [28,29,30]. Because rapidly evolving RNA viruses undergo antigenic drift, vaccine formulations often require periodic updating. 

In endemic areas, outbreaks may be caused by different strains or serotypes, sometimes circulating simultaneously, as occurs with bluetongue virus in ruminants and influenza viruses in humans. Consequently, multivalent vaccination is an established strategy for broadening protection against antigenically diverse arboviruses. For bluetongue virus, polyvalent vaccines have been used in endemic regions, and experimental multiserotype formulations have provided protection against multiple included serotypes in sheep and cattle [31,32]. Comparable findings in influenza and other arboviruses support evaluation of this strategy. Exposure to pandemic A(H1N1) 2009 broadened serological cross-reactivity to other swine influenza viruses, while multivalent dengue vaccines elicited neutralizing antibodies against all included serotypes without detectable immune interference [33,34].

The use of BEFV vaccines incorporating viruses from distinct lineages may also provide better protection from possible infection by antigenically divergent BEFV strains. As shown in Figure 3a, vaccinated animals exhibited neutralizing antibody titers against the homologous strain 919 that were approximately four-fold higher than those against the 2023 ‘Mayotte-like’ strain. Among unvaccinated animals exposed to the 2021 BEFV strain, this difference was even greater, with neutralizing titers against the homologous 2021 strain being approximately 6.5-fold higher than those against the 2023 ‘Mayotte-like’ strain (Figure 3b). In contrast, among vaccinated animals subsequently exposed to the 2021 BEFV strain, neutralizing antibody titers against both the 2021 BEFV strain and strain 919 were only approximately 2.3-fold higher than those against the 2023 ‘Mayotte-like’ strain. Furthermore, among vaccinated animals exposed to the 2023 strain, between-strain differences were generally smaller: four of the six pairwise comparisons were not statistically significant, although titers against the vaccine strain and the 2021 strain remained significantly higher than those against the 2000 strain (Figure 3e; Table 3). These findings should be interpreted with caution, as some of the serum samples were obtained from mature cows that may have been previously exposed to other BEFV circulating strains. This is of less concern for the vaccinated animals exposed during the 2021 outbreak because the virus responsible for the 2018 outbreak was highly similar to the 2021 strain [4]. In contrast, animals sampled following the 2023 outbreak may also have been exposed during the 2021 outbreak, which could account for the similar neutralizing antibody titers observed against all virus strains. Taken together, our findings suggest that sequential exposure to antigenically distinct BEFV strains may significantly broaden the neutralizing antibody response and enhance protection against heterologous viruses. Though our study points to a significant difference between the Mayotte-like strain and the vaccine strain, it should be remembered that in vitro neutralization indicates antigenic matching but does not directly indicate clinical protection; therefore, challenge experiments or prospective field-effectiveness studies are required to determine the protective efficacy of the current vaccine against the emerging Mayotte-like strain.

## 5. Conclusions

This study highlights the continuing challenges of controlling BEF through vaccination, particularly following the emergence of antigenically distinct strains such as the ‘Mayotte-like’ lineage I strain. Although the current Ultravac^®^ vaccine has demonstrated dose-dependent intermediate-term effectiveness against previously circulating strains, the substantially lower neutralization of the ‘Mayotte-like’ strain by vaccine-elicited sera indicates a marked antigenic mismatch and raises concern that protection against this emerging lineage may be reduced. To fully address this concern, prospective field-effectiveness studies or controlled challenge trials are required to determine whether this mismatch translates into reduced clinical protection. Given the continued circulation and emergence of antigenically divergent BEFV strains and their potential for geographic spread, the development and evaluation of updated vaccine candidates—particularly multivalent formulations designed to broaden protection against circulating and newly emerging strains—should be prioritized in parallel.

## Figures and Tables

**Table 1:** Information on age, vaccine, and exposure status of the cattle included in the study.

Group of Sera	Collection Date	№ of Animals (Calves & Cows)	Vaccination	Exposed 2021	Exposed 2023
vac-21	aug-sep-21	17	yes	no	No
exp-21	aug-sep-21	16	no	yes	No
vac&exp-21	aug-sep-21	17 (9 & 8) *	yes	yes	no
exp-23	jun-dec-23	14 (1 & 13) *	no	unknown	yes
vac&exp-23	jun-dec-23	23 (2 & 21) *	yes	unknown	yes

* Total number of animals and in brackets—number of calves & number of cows; vac—sera from vaccinated animals unexposed to natural infection by BEFV; exp-21—sera from non-vaccinated animals exposed to BEFV during 2021; vac&exp-21 sera from vaccinated animals exposed to BEFV during 2021; exp-23—sera from non-vaccinated animals exposed to BEFV in 2023; vac&exp-23—sera from vaccinated animals exposed to BEFV in 2023.

**Table 3:** Statistical summary of paired differences in log2 serum-neutralization titers among different BEFV strains, analyzed using a paired-samples *t*-test for normally distributed data and by the two-sided Wilcoxon signed-rank test for non-normally distributed data. AUS, Australian vaccine strain 919; ISR_2000, Israeli 2000 strain (Yakum-2000); ISR_2021, Israeli 2021 strain; ISR_2023, Israeli 2023 strain (Mayotte-like). Vac-21—animals vaccinated by Ultravac^®^ in 2021; Exp-21—non-vaccinated animals exposed to BEFV during 2021; Vac&Exp-21—animals vaccinated by Ultravac^®^ and exposed to BEFV during 2021; Vac&Exp-23—animals vaccinated by Ultravac^®^ and exposed to BEFV during 2023; Exp-23—non-vaccinated animals exposed to BEFV during 2023; S.E.—standard error; CI—confidence interval of 95%; *p*-values—statistical significance from paired *t*-tests.

Group of Sera	Paired Strains	Mean Diff.	S.E.	№ of Tested Sera	Lower CI	Upper CI	*p*-Value
Vac-21	AUS vs. ISR_2000	1.06	0.22	17	0.59	1.53	<0.01 *
	AUS vs. ISR_2021	0.59	0.31	17	−0.07	1.25	0.08
	AUS vs. ISR_2023	2.03	0.26	17	1.47	2.59	<0.01
	ISR_2000 vs. ISR_2021	−0.47	0.19	17	−0.88	−0.06	0.03
	ISR_2000 vs. ISR_2023	0.97	0.21	17	0.52	1.42	<0.01
	ISR_2021 vs. ISR_2023	1.44	0.22	17	0.97	1.91	<0.01
Exp-21	AUS vs. ISR_2000	0.03	0.26	16	−0.52	0.59	0.91
	AUS vs. ISR_2021	−1.75	0.31	16	−2.41	−1.09	<0.01
	AUS vs. ISR_2023	0.94	0.24	16	0.43	1.44	<0.01
	ISR_2000 vs. ISR_2021	−1.78	0.27	16	−2.36	−1.2	<0.01 *
	ISR_2000 vs. ISR_2023	0.91	0.19	16	0.5	1.31	<0.01
	ISR_2021 vs. ISR_2023	2.69	0.19	16	2.29	3.09	<0.01 *
Vac&Exp-21	AUS vs. ISR_2000	0.79	0.19	17	0.39	1.2	<0.01 *
	AUS vs. ISR_2021	0.06	0.21	17	−0.39	0.5	0.4 *
	AUS vs. ISR_2023	1.24	0.4	17	0.39	2.08	<0.01
	ISR_2000 vs. ISR_2021	−0.74	0.23	17	−1.23	−0.24	<0.01
	ISR_2000 vs. ISR_2023	0.44	0.33	17	−0.25	1.13	0.20
	ISR_2021 vs. ISR_2023	1.18	0.35	17	0.44	1.91	<0.01
Exp-23	AUS vs. ISR_2000	−0.11	0.35	14	−0.87	0.65	0.77
	AUS vs. ISR_2021	−1.21	0.42	14	−2.12	−0.31	0.01
	AUS vs. ISR_2023	−2.25	0.52	14	−3.38	−1.12	<0.01
	ISR_2000 vs. ISR_2021	−1.11	0.34	14	−1.83	−0.38	<0.01 *
	ISR_2000 vs. ISR_2023	−2.14	0.49	14	−3.2	−1.09	<0.01
	ISR_2021 vs. ISR_2023	−1.04	0.25	14	−1.57	−0.5	<0.01
Vac&Exp-23	AUS vs. ISR_2000	0.78	0.19	23	0.39	1.18	<0.01
	AUS vs. ISR_2021	0.17	0.26	23	−0.36	0.71	0.51
	AUS vs. ISR_2023	0.33	0.31	23	−0.31	0.96	0.3
	ISR_2000 vs. ISR_2021	−0.61	0.26	23	−1.16	−0.06	0.045 *
	ISR_2000 vs. ISR_2023	−0.46	0.27	23	−1.02	−0.11	0.11
	ISR_2021 vs. ISR_2023	0.15	0.17	23	−0.20	0.5	0.37

* Non-normally distributed data were analyzed by the two-sided Wilcoxon signed-rank test.

## Data Availability

Data will be available upon request.

## Supplementary Materials

The following supporting information can be downloaded at: https://www.mdpi.com/article/10.3390/vaccines14090830/s1, **Figure S1**: Paired differences in serum-neutralization titers among bovine ephemeral fever virus strains. Each blue point represents the within-animal paired difference in log_2_ serum-neutralization titer, calculated as the titer against the first named strain minus the titer against the second named strain. Points were horizontally jittered solely to improve visibility. Red circles indicate the mean paired difference, and black error bars represent the standard error (SE). Two-sided paired-samples *t*-test *p*-values and mean differences are displayed above each comparison. The horizontal dashed line at zero indicates no difference between the paired titers. The serum groups were: (a) vaccinated animals without recorded natural exposure sampled in 2021 (Vac-21); (b) unvaccinated animals clinically affected during the 2021 outbreak (Exp-21); (c) vaccinated animals clinically affected during the 2021 outbreak (Vac&Exp-21); (d) unvaccinated animals clinically affected during the 2023 outbreak (Exp-23); and (e) vaccinated animals clinically affected during the 2023 outbreak (Vac&Exp-23). AUS, Australian 919 vaccine strain; ISR_2000, Israeli Yakum-2000 strain; ISR_2021, Israeli ISR-3180/1/21 strain; ISR_2023, Israeli Mayotte-like ISR-1512/23 strain; BEFV, bovine ephemeral fever virus; SE, standard error. **Table S1**: List of primers used for partial sequencing of “Mayotte-like” strains of bovine ephemeral virus.
