# Supplementary material for: Low Match Between the Emerging ‘Mayotte-like’ Strain and the 919-Strain Bovine Ephemeral Fever Vaccine
Source: Vaccines (Basel). 2026 Sep 21;14(9):830. doi: 10.3390/vaccines14090830 (PMC13612049; doi:10.3390/vaccines14090830)
Supplement: Supplementary file 1 [file vaccines-14-00830-s001.zip › vaccines-4553093-supplementary.pdf]

Table S1. List of primers used for partial sequencing of "Mayotte-like" strains of bovine ephemeral virus.

name	Oligo sequence (5′ to 3′)	length of product	source
BEFma-36F	ACC CAA TTA GTC TTT CAA CAG GT	714	this study
Eph-N-727R	GCW GCT GCA TCY TTR TAT CTR GA		[19]
Eph-N-451F	GAT GAA AMW GAT GAY KTA TGG TTA A	992	[19]
BEFma-1420R	TTA TCT TGG GTT TCA ATT GG		this study
BEFma-1311F	GAA AAT TCA GAA TGC ACG TCC T	1034	this study
BEFma-2324R	GGA CTT CCC CTT CTT GAA AAG		this study
BEFma-2093F	GAC AAT ATG CCA GGA ACA TA	1099	this study
BEFma-3224R	CTA CAA ATA AAT CCT TCC AGA TGA		this study
BEFma-G62F	ATG TTC CGG TGA ATT GTG G	956	this study
BEFma-G999R	TGC ATA ATC TCT TCC TGG T		this study
BEFma-G771F	AGA ATG GTG GAG TAT AGA AAA CC	1038	this study
BEFma-G1785R	TCG TAT AAC CCT AAT TCT AAA TCC		this study

Figure S1a. Vac-21 serum group (n = 17)

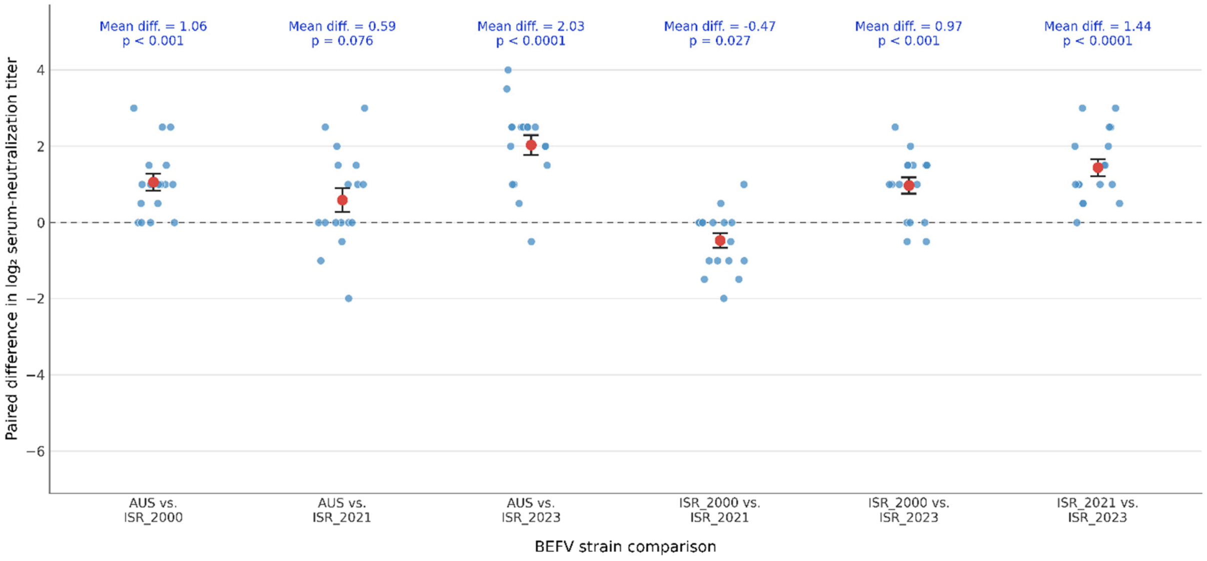

Figure S1b. Exp-21 serum group (n = 16)

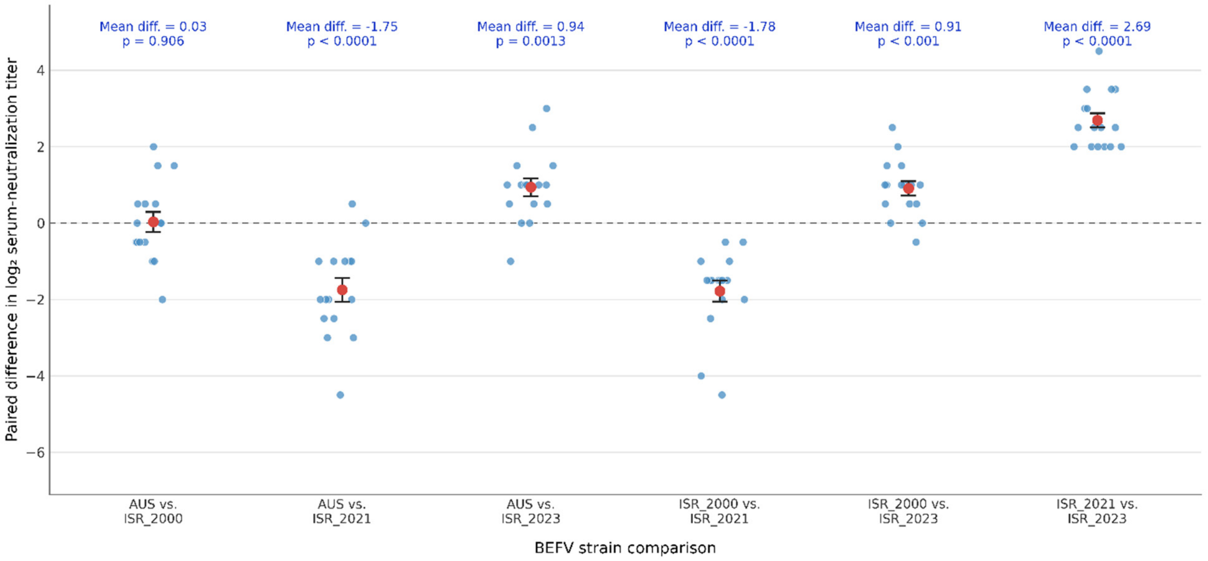

Figure S1c. Vac&Exp-21 serum group (n = 17)

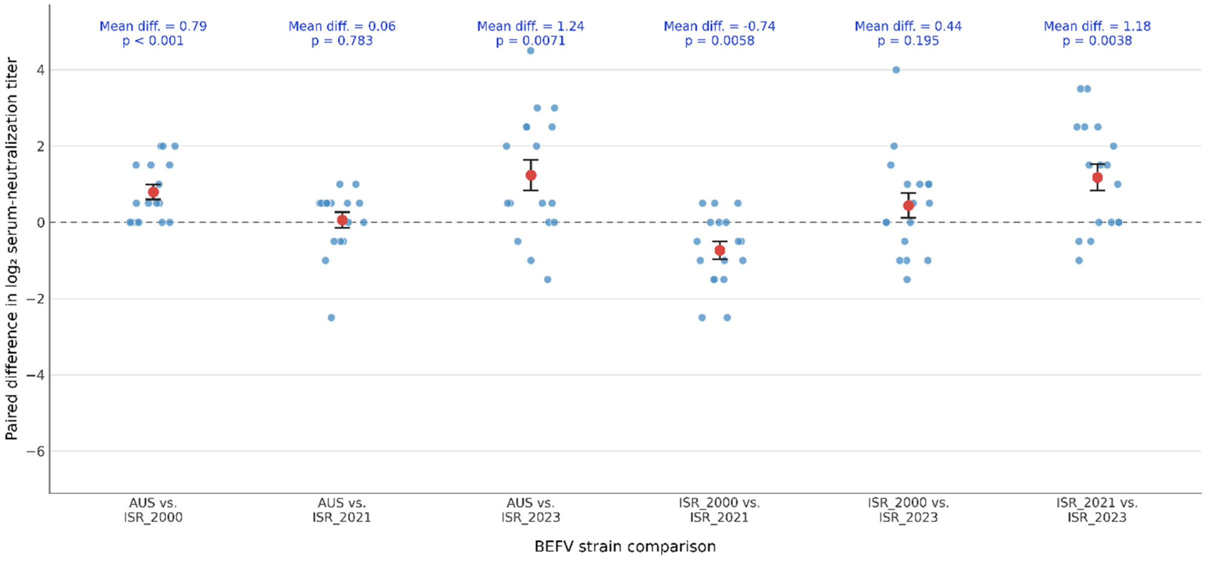

Figure S1d. Exp-23 serum group (n = 14)

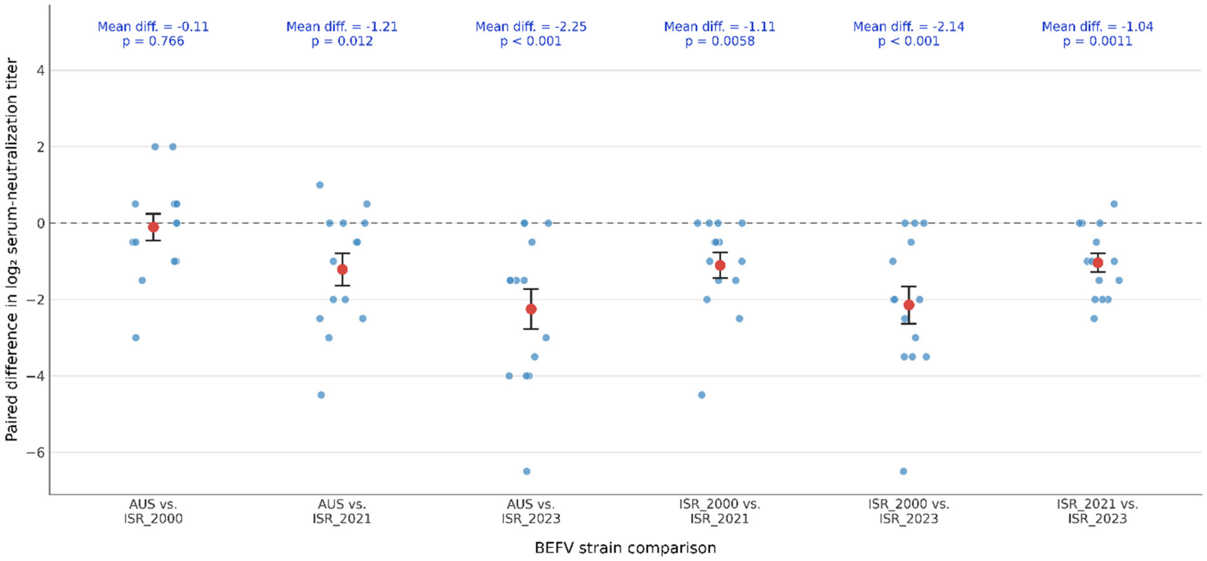

Figure S1e. Vac&Exp-23 serum group (n = 23)

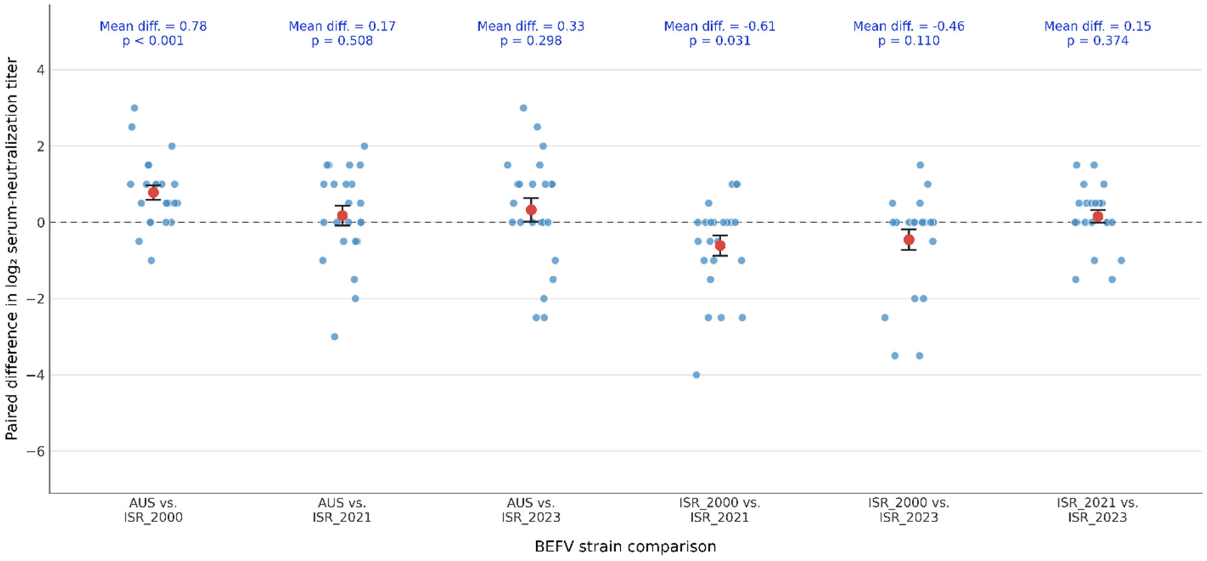

Figure S1. a-e: Paired differences in serum-neutralization titers among bovine ephemeral fever virus strains. Each blue point represents the within-animal paired difference in log_2 serum-neutralization titer, calculated as the titer against the first named strain minus the titer against the second named strain. Points were horizontally jittered solely to improve visibility. Red circles indicate the mean paired difference, and black error bars represent the standard error (SE). Two-sided paired-samples t-test p-values and mean differences are displayed above each comparison. The horizontal dashed line at zero indicates no difference between the paired titers.

The serum groups were: (a) vaccinated animals without recorded natural exposure sampled in 2021 (Vac-21); (b) unvaccinated animals clinically affected during the 2021 outbreak (Exp-21); (c) vaccinated animals clinically affected during the 2021 outbreak (Vac&Exp-21); (d) unvaccinated animals clinically affected during the 2023 outbreak (Exp-23); and (e) vaccinated animals clinically affected during the 2023 outbreak (Vac&Exp-23). AUS, Australian 919 vaccine strain; ISR_2000, Israeli Yakum-2000 strain; ISR_2021, Israeli ISR-3180/1/21 strain; ISR_2023, Israeli Mayotte-like ISR-1512/23 strain; BEFV, bovine ephemeral fever virus; SE, standard error.
